# Superior photothermal conversion performance of black titanium-based materials

**DOI:** 10.1016/j.isci.2025.112188

**Published:** 2025-03-10

**Authors:** Jun Li, Enhui Wu, Zhong Xu, Jing Hou, Wenjing Peng, Hong Li, Xiang Li

**Affiliations:** 1 Panzhihua University, Panzhihua 617000, China; 2 Vanadium and Titanium Resources Comprehensive Utilization Key Laboratory of Sichuan Province, Panzhihua 617000, China

**Keywords:** Materials application, Materials characterization, Materials science

## Abstract

The application of photothermal conversion technology in the fields of seawater desalination and wastewater treatment stands as a potent approach to alleviating the global water scarcity crisis. In this research, we have successfully synthesized black titanium-based photothermal conversion materials utilizing the aluminothermic reduction method. The experimental results demonstrate that the crafted black TiO_2_ exhibits a notable overall solar energy absorptivity of 65.7% and a photothermal conversion efficiency of 87.5%. Notably, the black Magneli phase titanium oxide (Ti_4_O_7_ and Ti_5_O_9_), derived from nanorutile TiO_2_, exhibits an even more impressive overall solar absorptivity of 83.4% and a photothermal conversion efficiency of 93.8%. Under a light intensity of 5 kW/m^2^, this material achieves an evaporation rate of 4.4 kg m^−2^·h^−1^ and an evaporation efficiency of 63%, underscoring its vast potential for applications in wastewater purification and seawater desalination.

## Highlights

• Aluminothermic synthesis of black Ti-based material under ambient vacuum-free conditions
• Broad-spectrum light absorption with 93.8% photothermal efficiency for solar applications
• Black Ti-based evaporator enables superior solar-driven water purification
• Black Ti-based film rivals commercial blue film in collector thermal performance

## Graphical abstract

**Figure:**
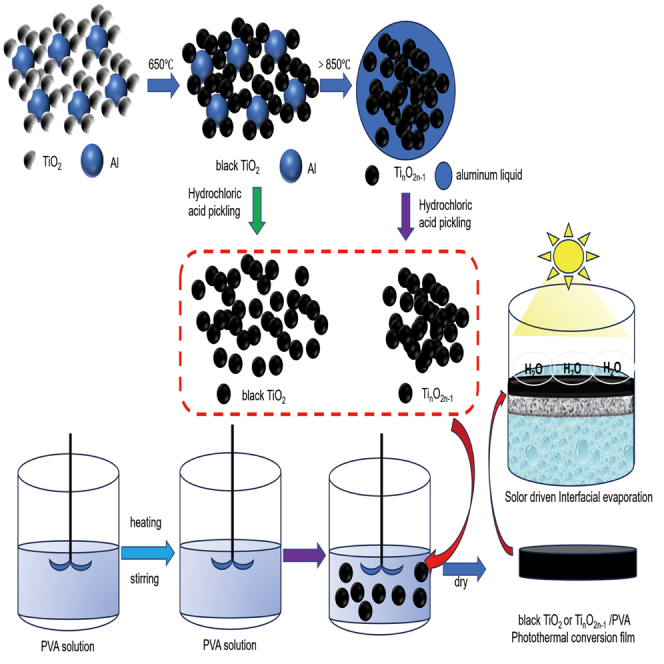

## Introduction

As water pollution and shortages, energy shortages, and environmental pollution become more severe, the search for clean energy is an urgent task. Solar energy has received significant attention from researchers due to its cleanliness, accessibility, and abundance of sources. Current solar energy research focuses on photothermal conversion,^1^^,^^2^photoelectric conversion,^3^^,^^4^ hydrogen production,^5^^,^^6^ photocatalytic oxidation,^7^^,^^8^ and water purification or distillation.^9^ Photothermal conversion technology is the most efficient way of using energy, designed to purify wastewater and desalinate seawater without additional energy input.

Photothermal materials are the focus of investigation as carriers of light absorption and energy conversion. The following three requirements must generally be met by ideal photothermal materials: first, they must be widely absorbing and encompass the whole solar spectrum; second, they must have low levels of emissivity to guarantee the maximum efficiency in converting light energy into heat; and third, they must be composed of widely accessible materials and economically scaled up for industrial production.^10^ Photothermal materials mainly include metal-based nanoparticles,^11^^,^^12^^,^^13^ carbon matrix composites,^14^ and organic compounds.^15^^,^^16^ Additionally, scientists both domestically and internationally are becoming increasingly interested in carbon-based composite materials doped with different micro-nano metal materials or semiconductors.^17^ In general, metal-based nanoparticles are more costly and exhibit subpar thermal stability. Carbon matrix composites must be prepared and functionalized under somewhat strict conditions. Photothermal materials made of organic compounds are susceptible to photodegradation. Thus, creating photothermal conversion materials with superior performance, low cost, and an easy synthesis process remains a challenge.

Titanium is the ninth element found on earth and its metal oxide compounds such as titanium dioxide have been extensively studied due to their photoreactive ability, low cost, and high thermal stability.^18^ However, TiO_2_ has a wide bandwidth (3 eV–3.2 eV) and only responds to UV light at wavelengths below 400 nm, making it unsuitable for solar-spectrum photothermal conversion.

Titanium nitride (TiN) NPs exhibit broad plasmonic resonance in the visible-NIR range so that they can harvest the majority of the solar spectrum. Kaur et al. prepared a series of titanium nitride based composite materials and conducted in-depth research in the fields of photocatalysis and photothermal conversion.^19^^,^^20^^,^^21^ A ceramic microfibrous solar steam generator was created by ceramic fiber wool (CW) and titanium nitride (TiN), which yields a solar thermal conversion efficiency of more than 80% at 1 kW/m^2^.^19^ TiN-AAO photothermal conversion material was prepared by loading titanium nitride nanoparticles (TiN NPs) onto anodized aluminum oxide (AAO), which steam generation efficiency reaches 92% under solar irradiation of 1 kW/m^2^.^21^

Chen et al. (2011) prepared black TiO_2_ nanoparticles with a narrow bandgap and excellent absorption properties in the full solar spectrum using hydrogen reduction, which can be used as a photothermal conversion material.^22^ Currently, chemical reduction,^23^^,^^24^ chemical oxidation,^25^ and electrochemical reduction^26^ have also been developed for the preparation of black TiO_2_. Ozin et al.^27^ prepared different black titanium metal oxides (mixtures) using Mg reduced TiO_2_. The water evaporation rate could reach up to 0.8 kg m^−2^·h^−1^ under the irradiation of 1 kW m^−2^ simulated light source, corresponding to an efficiency of 50.3%.

The Magneli phase Ti_n_O_2*n*-1_ has been shown to absorb a wide range of UV-visible light and is a promising material for photothermal conversion. Chen et al. investigated the photothermal conversion performance of submicron Ti_2_O_3_ materials, which reached 92.5% light absorption in the full spectral range and the evaporation rates of 5.03 kg m^−2^·h^−1^ at 5 kW m^−2^ solar irradiance.^10^ Yang Bo et al. investigated the photothermal conversion performance of λ-Ti_3_O_5_ material, which presented a 96.4% light absorption in the full solar spectral range and its water evaporation efficiency was as high as 6.09 kg m^−2^·h^−1^ at 1 solar illumination condition (kW·m^−2^).^28^

In this paper, we propose the preparation of black titanium-based materials through an aluminothermic reduction process. Black TiO_2_ and the Magneli phase Ti_n_O_2*n*-1_ were synthesized by carefully regulating the parameters of the reduction process, and a systematic investigation into the mechanism underlying this reduction was conducted. We thoroughly examined both the light absorption capacity and photothermal conversion efficiency of the resulting black titanium-based materials. Furthermore, we explored the correlation between solar water vapor generation efficiency and various experimental parameters in relation to practical applications in seawater desalination. Additionally, we investigated the potential use of these materials as heat-absorbing substances in flat plate collectors. The findings presented in this paper will contribute to advancing desalination techniques and solar thermal conversion for large-scale industrial applications.

## Results and discussion

### Phase structure analysis of the sample

**Figure 1:**
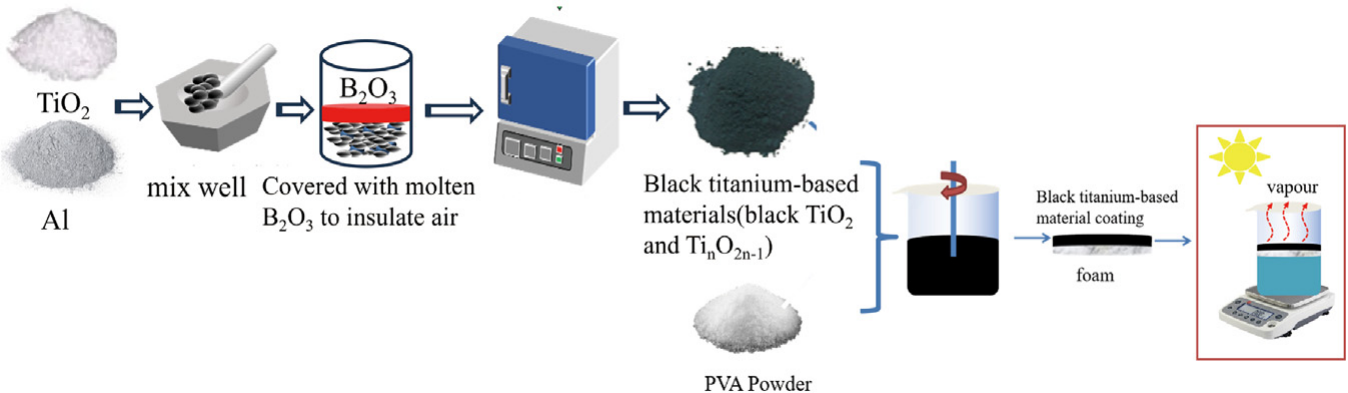
Schematic diagram of preparation of black titanium-based materials and photothermal conversion coatings

**Figure 2:**
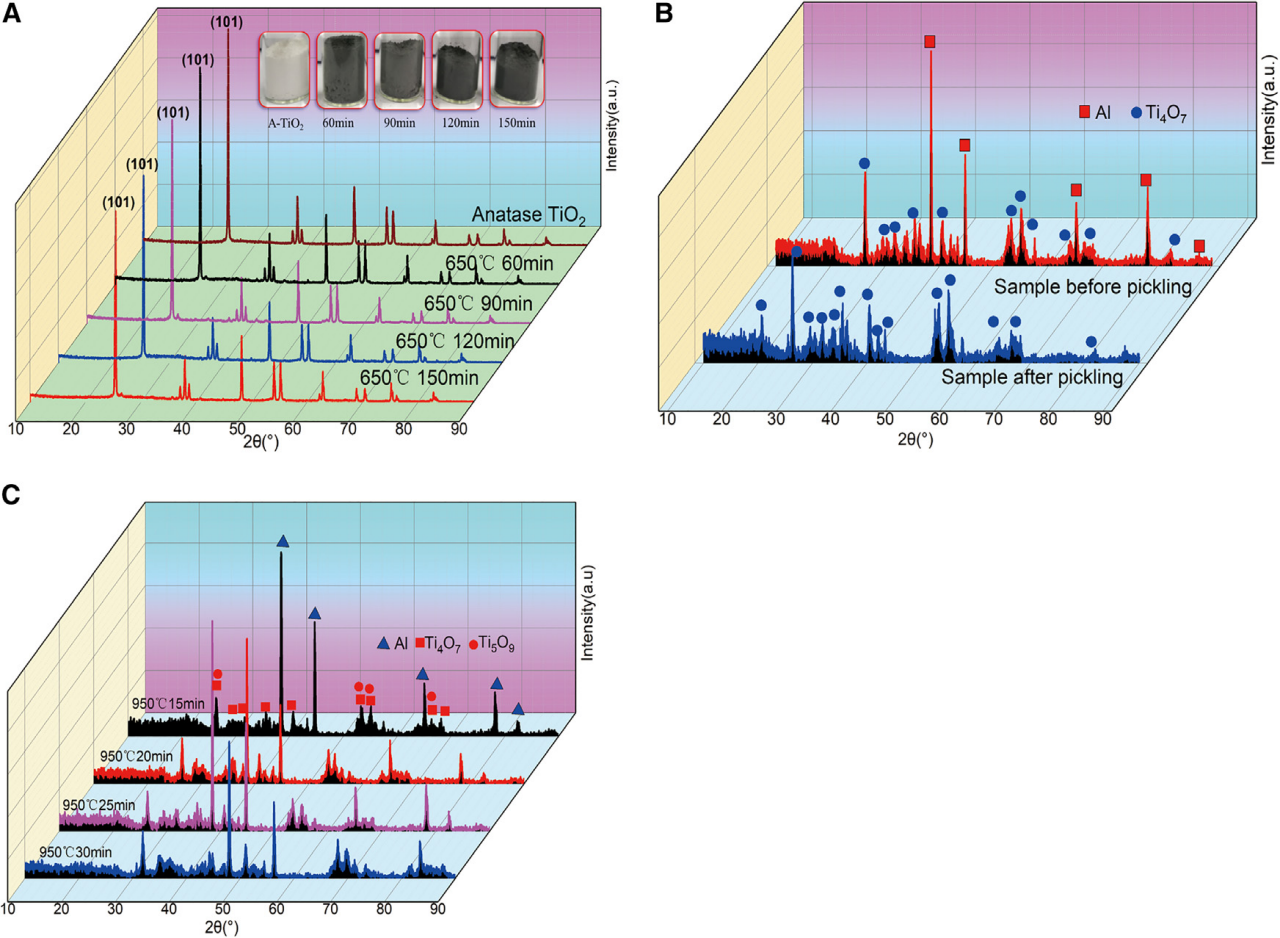
XRD analysis of samples under different process conditions (A) XRD analysis of samples prepared from anatase TiO_2_ as raw material at 650°C for different roasting times after hydrochloric acid pickling; (B) XRD analysis of samples prepared from anatase TiO_2_ as raw material before and after hydrochloric acid pickling by roasting at 950°C for 30 min; (C) XRD analysis of samples prepared from rutile TiO_2_ as raw material at 950°C for different roasting times.

Schematic diagram of the preparation of black titanium-based materials and photothermal conversion coatings is shown in Figure 1. The impact of various roasting durations and temperatures on the physical phases of the prepared samples was systematically examined, maintaining a constant mass ratio of Al powder to TiO_2_ powder at 0.2. As illustrated in Figure 2A, when anatase titanium dioxide and Al powder are utilized as raw materials and the roasting time exceeds 60 min, the color of white anatase TiO_2_ transforms to grey-black at a temperature of 650°C. XRD analysis reveals that the phase structure of the resultant black TiO_2_ remains consistent with that of white TiO_2_. However, in comparison to the raw material (anatase TiO_2_), the (101) plane diffraction peak of black TiO_2_ shifts to the right at smaller diffraction angles, and this shift increases with prolonged sintering time, potentially due to the introduction of lattice defects.^29^ As shown in Figure 2B, the color of the sample changes to deep black and its physical phase transforms to Ti_4_O_7_ as the roasting temperature rises to 950°C and the roasting time of 30 min. Hydrochloric acid pickling can effectively remove the excess Al and the reaction generated Al_2_O_3_ in the sample, a relatively pure sample with Ti_4_O_7_ as the main physical phase can be obtained after the pickling.

To investigate the influence of titanium dioxide with different crystal structures and particle sizes on the physical phase of the prepared samples, nano-sized rutile titanium dioxide was employed as the raw material. As illustrated in Figure 2C, the as-prepared samples exhibited a blue-black color, and their primary physical phase consisted of a mixture of Ti_4_O_7_ and Ti_5_O_9_ when roasted at 950°C for 15–30 min.

### Mechanism analysis of black titanium-based materials prepared by aluminum thermal reduction method

**Figure 3:**
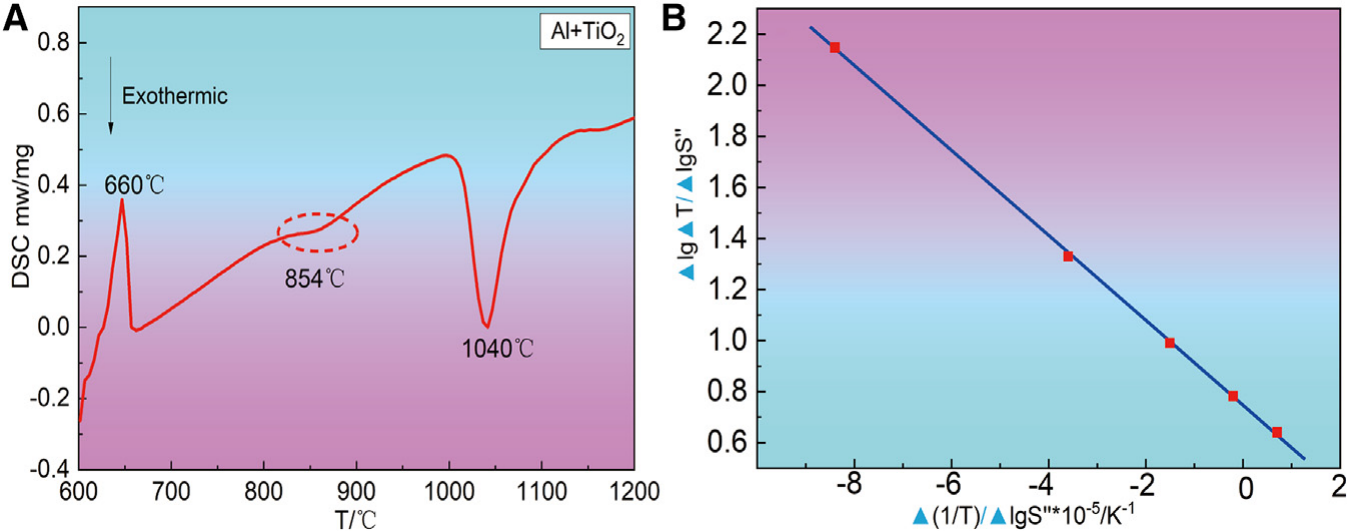
Thermal characteristics and reaction kinetics analysis of the Al+TiO₂ reaction system (A) DSC curves of the Al-TiO_2_ system; (B) Freeman-Carroll curves of the exothermic fronts were plotted in conjunction with the classical kinetic equations.

Based on the DSC curves of the Al-TiO_2_ system determined in Figure 3A, the Freeman-Carroll curves of the exothermic fronts were plotted in conjunction with the classical kinetic equations, as shown in Figure 3B. The curve equation fitted according to Figure 3B is Y = −16216X+0.415; Therefore, E_a_*/*2.303R = 16216; the activation energy (E_a_) of the Al-TiO_2_ system is 310.5 kJ mol^−1^, which is closer to the diffusion activation energy of O in TiO_2_. It indicates that the Al-TiO_2_ reaction is controlled by the diffusion of O in TiO_2_. The kinetic equation for the reaction between Al and TiO_2_ is given by reaction (1). The kinetic equation shows that the reaction rate is proportional to the temperature. The rate of reaction accelerates as the temperature increases.

(Equation 1) $$\frac{d\alpha }{dT}=\frac{A}{ø}{\left(1-\alpha \right)}^{0.415}\;\exp\left(-\frac{310.5}{RT}\right)$$

**Figure 4:**
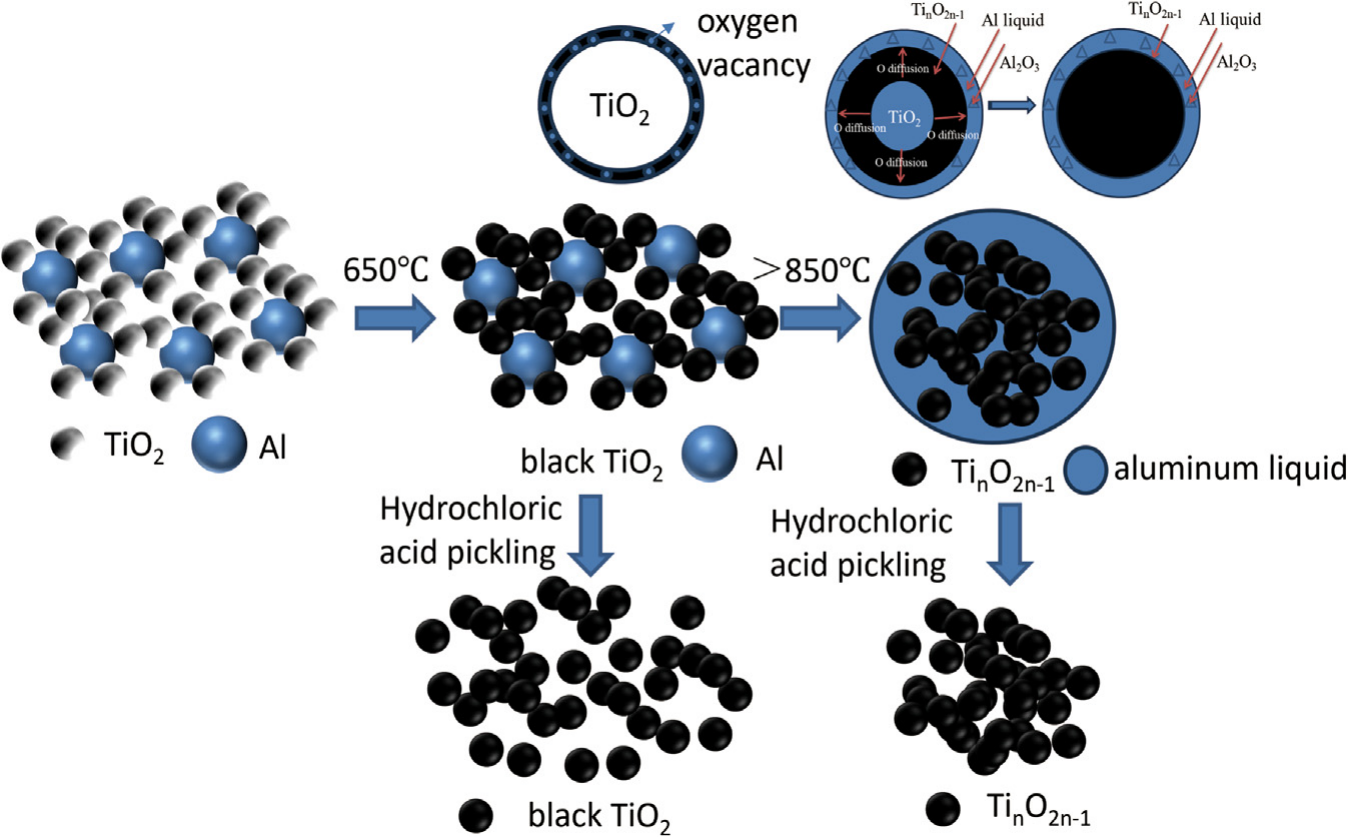
Schematic diagram of macro reaction of aluminum reduction TiO_2_

Figure 4 shows the possible macroscopic reaction process of Al reduction of TiO_2_ can be inferred combining DSC analysis and XRD analysis of samples prepared under different temperature conditions. The main steps are as follows:(1) Large Al particles are encapsulated by fine TiO_2_ particles after the raw materials are mixed well. (2) Due to the higher reducibility of Al than Ti, O starts to diffuse from the inside of TiO_2_ to the surface of TiO_2_ as the temperature reaches 650°C. O vacancies are formed on the surface of TiO_2_ crystals, and the color of TiO_2_ changes from white to black. (3) Al powder begins to melt into Al liquid and leach along the pores between the TiO_2_ particles piled up around it as the temperature continues to rise, the diffusion of O from the interior of TiO_2_ to the surface of TiO_2_ is accelerated, and the crystal structure changes to form a non-stoichiometric ratio of Ti_n_O_2*n*-1_(Ti_4_O_7_, Ti_5_O_9_). The reactions carried out at this stage are: Al+TiO_2_→Al_2_O_3_+Ti_n_O_2*n*-1_.

### Morphological and particle size analysis of samples

**Figure 5:**
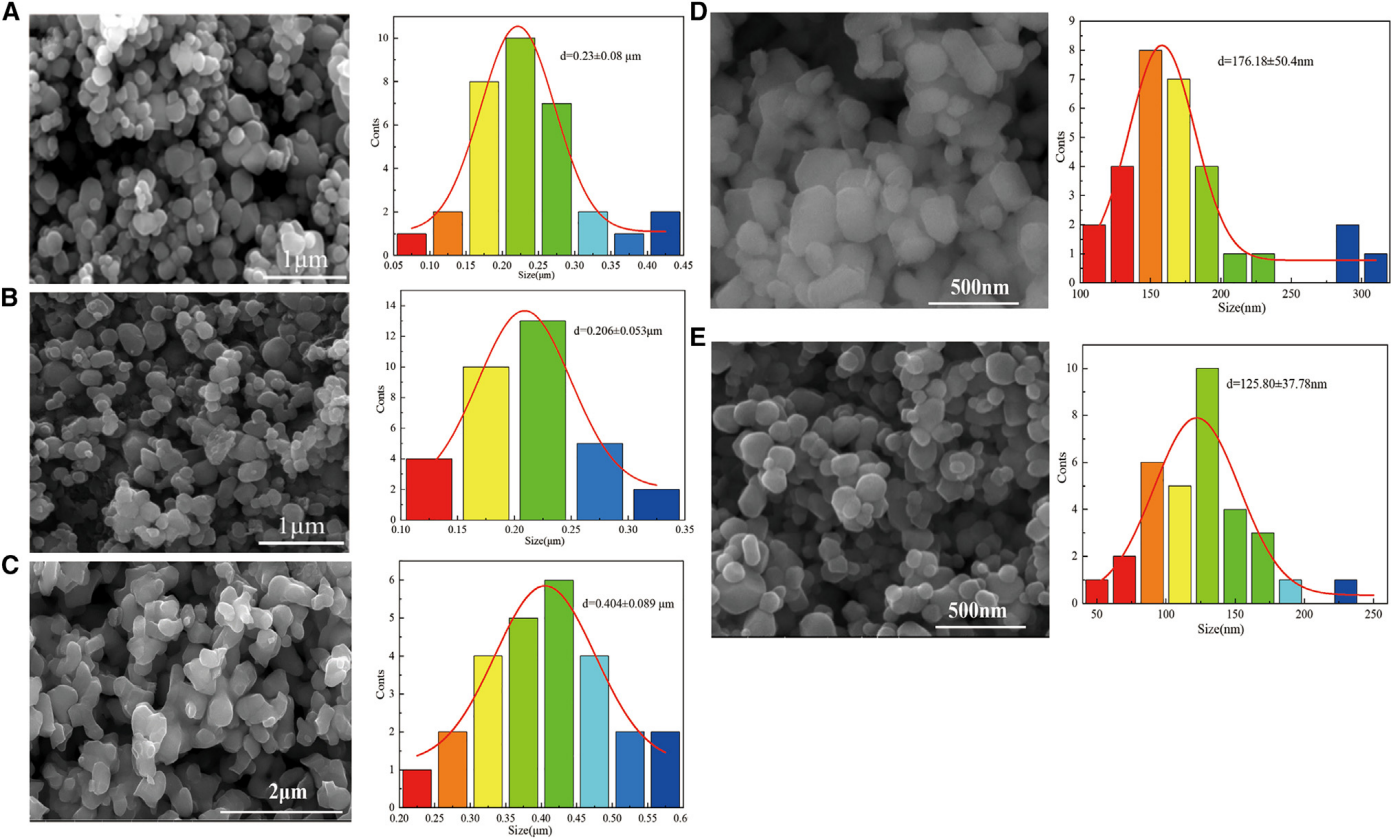
SEM analysis and particle size of raw materials and samples under different process conditions (A) Anatase titanium dioxide. Scale bar:1 μm; (B) Black TiO_2_ prepared from anatase titanium dioxide as raw material by roasting at 650°C for 120 min after acid leaching. Scale bar:1 μm; (C) Magnéli phase Ti_4_O_7_ material prepared from anatase titanium dioxide as raw material by roasting at 950°C for 30min after acid leaching. Scale bar:2 μm; (D) Rutile titanium dioxide raw material. Scale bar: 500 nm; (E) Magneli phase Ti_4_O_7_ and Ti_5_O_9_ prepared from rutile titanium dioxide as raw material by roasting at 950°C for 25min after acid leaching. Scale bar: 500 nm.

Figure 5 shows SEM analysis of raw materials and samples under different process conditions. As shown in Figures 5A–5C, the particle size of anatase titanium dioxide was 0.23 ± 0.08 μm. The particle size of black TiO_2_ obtained by acid leaching after roasting at 650°C for 120 min was 0.23 ± 0.053 μm, and there was almost no sintering growth compared to the raw material. With the increase of roasting temperature, the particle size of the sample prepared by acid leaching at 950°C for 30 min grew to 0.404 ± 0.089 μm, and there was obvious sintering cross-linking phenomenon. As depicted in Figures 5D and 5E), the particle size of rutile titanium dioxide was 176 ± 50.4 nm. The particle size of sample obtained by acid leaching after roasting at 950°C for 25 min was 125.8 ± 37.78 nm, and there was almost no sintering growth compared to the raw material. The nano-sized black titanium-based materials can be successfully prepared from nano-sized rutile titanium dioxide.

### X-Ray photoelectron spectroscopy (XPS) analysis of samples

**Figure 6:**
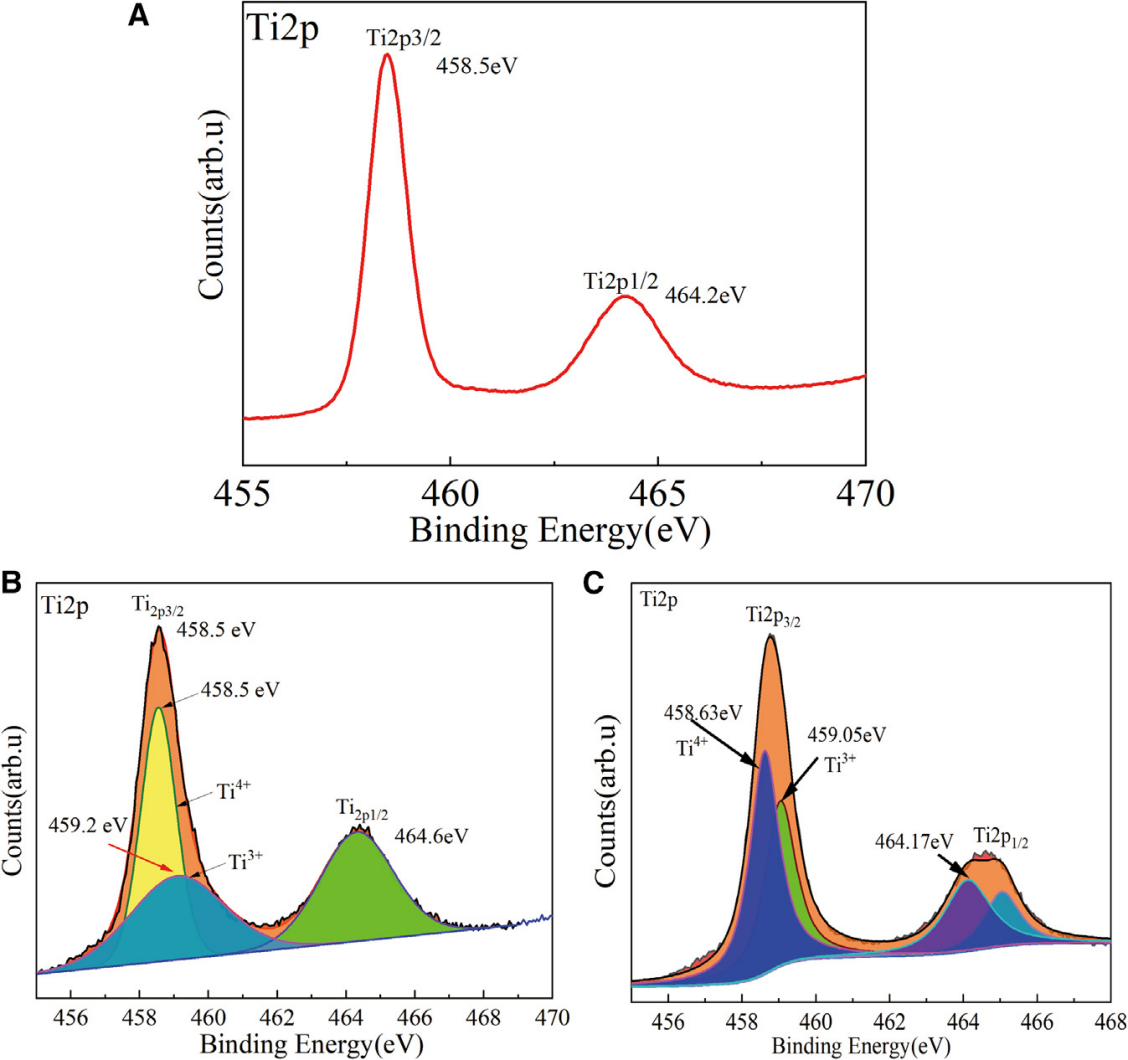
X-ray photoelectron spectroscopy (XPS) analysis of samples (A) Ti2p core level XPS spectra of TiO_2_; (B) Ti2p core level XPS spectra of Magneli phase Ti_4_O_7_ material prepared from anatase TiO_2_ at 950°C for 30 min; (C) Ti2p core level XPS spectra of Magneli phase (Ti_4_O_7_ and Ti_5_O_9_) material prepared from rutile TiO_2_ at 950°C for 25 min.

Figures 6A–6C exhibit the Ti2p core-level XPS spectra for TiO_2_, Magneli phase Ti_4_O_7_ material prepared from anatase TiO_2_ at 950°C for 30 min, and Magneli phase (Ti_4_O_7_ and Ti_5_O_9_) material prepared from rutile TiO_2_ at 950°C for 25 min, respectively. As observed in Figure 6B, the two broader peaks situated at approximately 458.5 eV and 464.6 eV correspond to the binding energies of Ti2p3/2 and Ti2p1/2, respectively. The Ti2p3/2 peak can be further deconvoluted into two distinct peaks at around 458.5 eV and 459.2 eV. The peak at 458.5 eV is associated with Ti^4+^ ions in rutile TiO_2_,^30^ while the peak at 459.2 eV is attributed to Ti^3+^ ions.

In Figure 6C, the Ti2p3/2 peak is similarly deconvoluted into two peaks at approximately 458.63 eV and 459.05 eV. The peak at 458.63 eV is related to Ti^4+^ ions in rutile TiO_2_, and the peak at 459.05 eV is indicative of Ti^3+^ ions. Notably, this peak exhibits tails in the region of lower binding energies, suggesting the presence of titanium ions in lower valence states. The crystalline structure of Magnéli Ti_4_O_7_ features rutile blocks interspersed with corundum-like Ti_2_O_3_ layers.^31^

### UV-Vis diffuse reflectance spectroscopy

**Figure 7:**
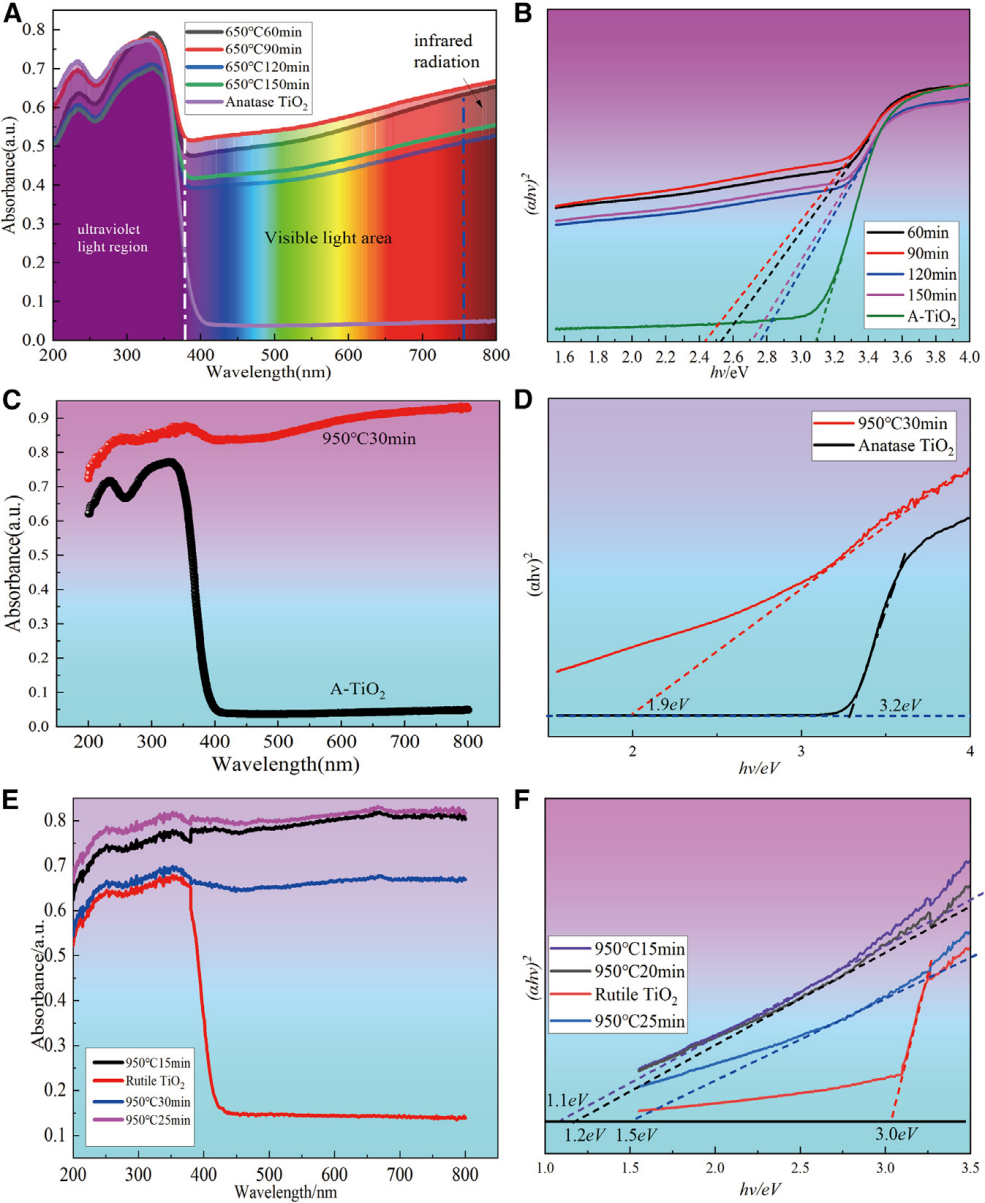
UV-visible absorption and the forbidden bandwidths of samples under different process conditions (A and B) black TiO_2_ prepared from anatase TiO_2_ as raw material by roasting at 650°C for 60–150 min; (C and D) Magneli phase Ti_4_O_7_ prepared from anatase TiO_2_ as raw material by roasting at 950°C for 30 min and (E and F) Magneli phase Ti_4_O_7_ and Ti_5_O_9_ prepared from rutile TiO_2_ as raw material by roasting at 950°C for 15 –30 min.

**Figure 8:**
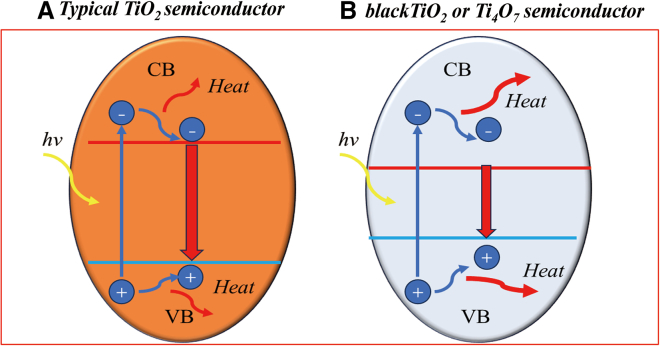
Schematic illustration of the generation and relaxation processes of electron-hole pairs in two different types of semiconductors Illustration of the electron-hole generation and relaxation in (A) a normal semiconductor(TiO_2_) and (B) a narrow-bandgap semiconductor(black TiO_2_ and Magneli phase Ti_n_O_2*n*-1_).

UV-visible absorption characterization was carried out in order to test the solar absorption properties of the samples prepared under different process conditions. Based on the UV-visible absorption spectra of the samples, the forbidden bandwidths of the corresponding samples were calculated. As depicted in Figures 7A and 7B), the black TiO_2_ prepared by roasting at 650°C for 60–150 min has a superior absorption performance in the UV region, visible region and near-infrared region, and the whole solar spectrum can reach 65.7% (Table 1). However, the white anatase titanium dioxide has a certain absorption performance in the ultraviolet region, but the absorption rate in the visible and near-infrared region is almost zero. Relative to the white anatase titanium dioxide, the forbidden bandwidth of the prepared black TiO_2_ narrows from 3.2 eV to 2.4–2.8 eV. As shown in Figures 7C and 7D, the whole solar spectrum of the sample prepared under the roasting condition at 950°C for 30 min was significantly increased to 91.3%(Table 1), and its forbidden bandwidth was further reduced to 1.9 eV. As shown in Figures 7E and 7F, the samples prepared from rutile titanium dioxide at 950°C for 15∼30min also showed good absorption performance, which total absorption capacity of 83.4% over the whole solar spectrum. The bandwidth of rutile titanium dioxide is 3.0eV, and the bandwidths of the samples prepared by roasting at 950°C for 15 min, 25 min, and 30 min are reduced to 1.1eV, 1.2eV, and 1.5eV. Figure 8 shows the schematically compares the photogenerated electron-hole pair generation and relaxation processes in conventional wide-band semiconductors and prepared narrow bandgap black titanium-based materials. In the prepared narrow bandgap black titanium-based material, a large number of electron-hole pairs above the bandgap are generated by sunlight irradiation due to the extremely narrow bandgap. These electrons and holes ubsequently relax to the band edge and convert the additional energy into heat through a thermalization process, instead of re-emitting photons through compounding as in broad-band semiconductors.

**Table 1:** Solar absorption properties of black titanium-based photothermal materials in different spectrum region prepared under different process conditions

Sample	Total	UV	Visible	Infrared
Solar	100%	7%	50%	43%
Anatase TiO_2_	5%	5%	0	0
Rutile	18%	5%	7%	6%
Black TiO_2_ prepared at 650°C for 90 min	65.7%	5.6%	35%	30.1%
Magneli phase Ti_4_O_7_ material prepared from anatase TiO_2_ at 950°C for 30 min	91.3%	6.3%	45%	40%
Magneli phase (Ti_4_O_7_ and Ti_5_O_9_) material prepared from rutile TiO_2_ at 950°C for 25 min	83.4%	5.7%	41%	35.7%

In summary, the prepared black titanium-based materials have a narrow-band system and excellent light absorption properties in the full spectral range, which can be used as high-efficiency solar photothermal conversion materials.

### Photothermal conversion properties of black titanium-based materials

Given their exceptional absorption capability, the prepared black titanium-based materials hold great promise for achieving the high photothermal efficiency as anticipated. The external photothermal conversion efficiency (η_ext_) is quantified by the ratio of the heat generated (Q) to the irradiation energy of the photon flux (q):

(Equation 2) $$\eta_{ext}=\frac{Q}{q}$$

We utilized a refined photocalorimetric method to ascertain the heat (Q) generated by our samples when exposed to an irradiation photon flux of 5 kW m^−2^ produced by a solar simulator. Under solar light irradiation, a material undergoes a temperature increase from room temperature (*T*_*surr*_) to a maximum temperature (*T*_*max*_). At this maximum temperature, the heat (*Q*) generated by the material is equivalent to the heat (*Q*_*surr*_) dissipated to the surrounding:

(Equation 3) $${Q=Q}_{surr}=h\times s\times \left(T_{\max}-T_{surr}\right)$$

where *h* refers to the heat transfer coefficient, and *s* is the surface area of the material for heat dissipation.

**Figure 9:**
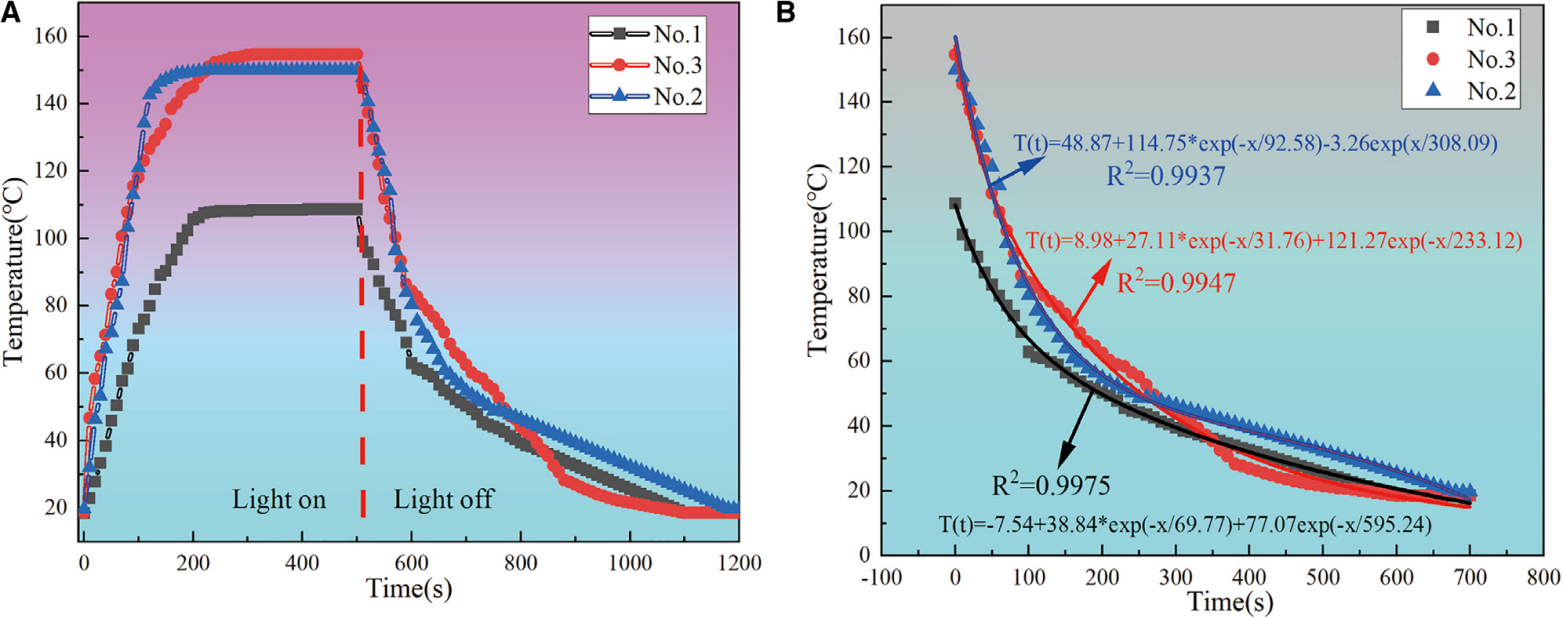
Temperature dynamics of black titanium-based material under irradiation and post-irradiation cooling (A) A typical temperature-time relationship showing the increase in the temperature of the as-prepared black titanium-based material under irradiation from 5 kW m^−2^ and the decay trace of temperature after the irradiation is turned off; (B) Cooling curve function is fitted according to the cooling curve.

The black titanium-based materials prepared under different conditions were pressed into discs and tested for heating and cooling under a 5 kW m^−2^, and the results are shown in Figure 9. The corresponding numbers of the different titanium-based materials are given in Table 2.

**Table 2:** The corresponding sample number of the different titanium-based materials

Number	Process conditions
No.1	Black TiO_2_ prepared from anatase TiO_2_ at 650°C for 90 min
No.2	Magneli phase Ti_4_O_7_ material prepared from anatase TiO_2_ at 950°C for 30 min
No.3	Magneli phase (Ti_4_O_7_ and Ti_5_O_9_) material prepared from rutile TiO_2_ at 950°C for 25 min

As shown in Figure 9A, we determined the *T*_*max*_*-T*_*surr*_ of No.1, No.2 and No.3 samples to be ≈ 89.8°C, 130.3°C and 136°C. The cooling curve function can be fitted according to the function in Equation 4,^10^ as shown in Figure 9B. The value of *w* can be derived from the cooling curve function, as shown in Equations 5 and 6.

(Equation 4) $$T\left(t\right)=A\times \exp\left(-wt\right)+B\times \exp\left(-w_{surr}t\right)+C$$

(Equation 5) $$T\left(t\right)=38.84\times \exp\left(-\frac{1}{69.77}t\right)+77.07\times \exp\left(-\frac{1}{595.24}t\right)-7.54$$

(Equation 6) $$T\left(t\right)=114.75\times \exp\left(-\frac{1}{92.58}t\right)-3.26\times \exp\left(\frac{1}{308.09}t\right)+48.87$$

(Equation 7) $$h\times s=w\times c_{i}\times m_{i}$$

*c*(black TiO_2_)≈c(TiO_2_) = 0.694 J/g°C,^32^ *c*(Ti_4_O_7_) = 0.68 J/g°C,^33^ *m*_i_ = 5g, *s* = 0.00102 m^2^; The value of *h×s* was found to be ≈ 48.7 mW °C^−1^ and 36.72 mW °C^−1^ by fitting the temperature cooling stages of the No.1 and No.3 with an exponential decay function. The external solar-heat conversion efficiency *η*_*ext*_ was calculated to be 87.5% (No.1) and 93.8% (No.3) according to Equations 2 and 3.

### Photothermal water evaporation performance of black titanium-based materials

**Figure 10:**
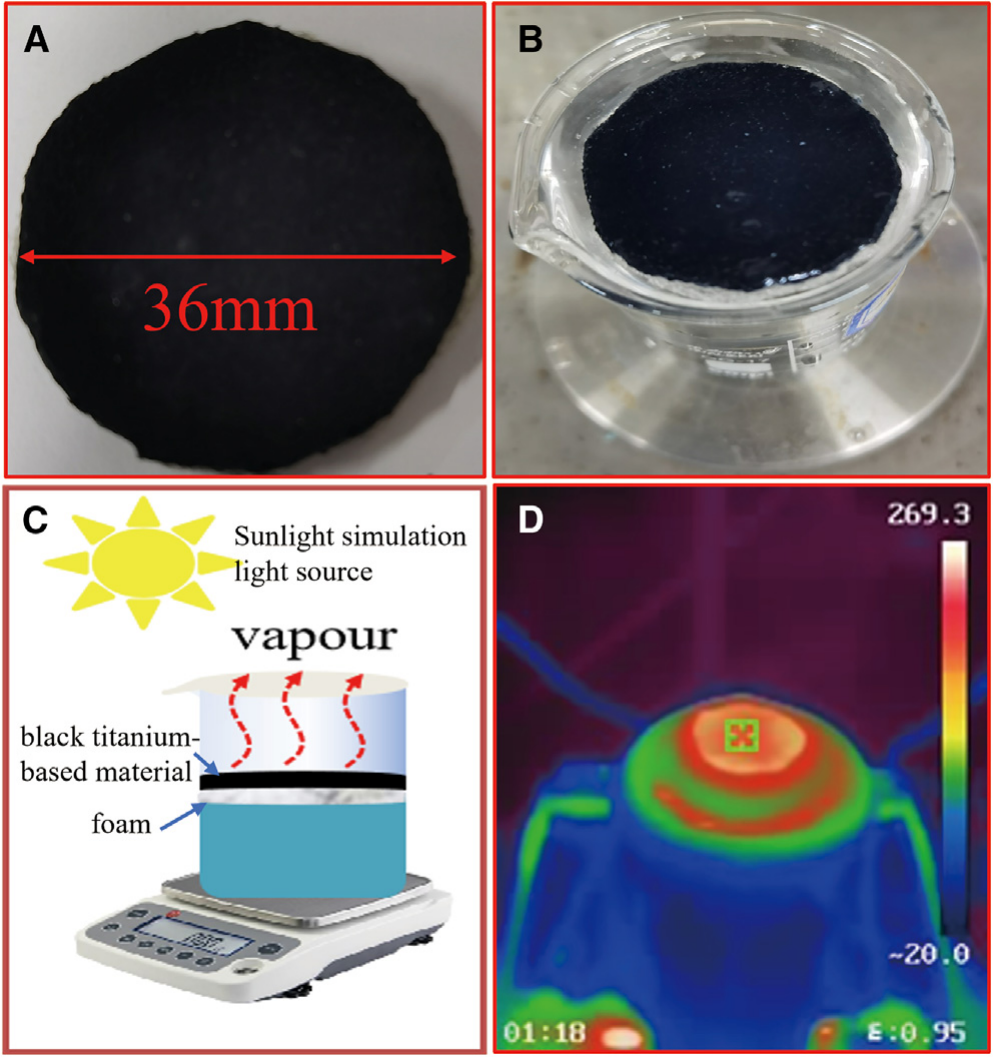
Solar-driven water evaporation system with black titanium-based material (A and B) Photothermal conversion coatings prepared from black titanium-based materials. (C) A schematic diagram of the solar water steam evaporation design where the foam was used to load the as-prepared black titanium-based material. (D) IR photograph of the water steam generated under solar illumination of 5 kW m^−2^.

To evaluate the photothermal water evaporation efficacy of the synthesized black titanium-based materials, we undertook an interfacial water evaporation analysis. As illustrated in Figure 10, this analysis scrutinized the steam evaporation performance of the black titanium-based materials across diverse process parameters under solar irradiation conditions of 5 kW m^−2^.

**Figure 11:**
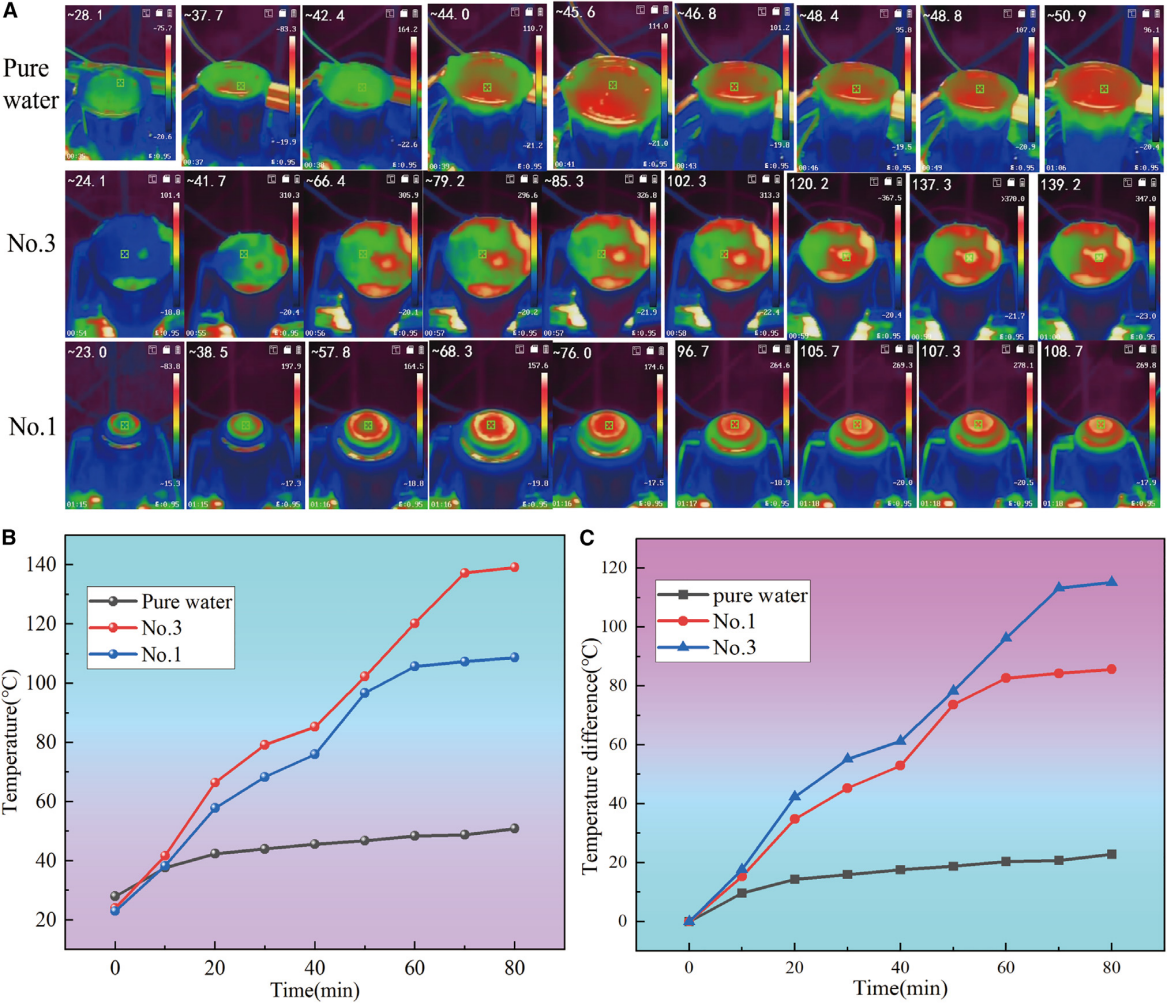
Solar irradiation effects on black titanium-based material in water: IR imaging, temperature dynamics, and gradient analysis (A) IR photograph of black titanium-based material thin film on top of the water surface; (B) Temperature change of black titanium-based material thin film on top of the water surface; (C) Temperature difference between the top water surface and the bottom section in the beaker upon solar light irradiation 5 kW m^−2^.

As illustrated in Figure 11A, when exposed to the intense radiation of a sunlight simulator set at 5 kW m^−2^, the surface temperatures of the interfacial evaporators corresponding to samples No.3 and No.1 soar to 140°C and 110°C, respectively, whereas the surface temperature of pure water remains significantly lower at 50°C. Additionally, Figure 11B reveals that the presence of the photothermal conversion film device results in a remarkable increase in water surface temperature by 85.7°C and 115.2°C for the respective samples. Conversely, in the absence of the photothermal conversion film device, the temperature rise is a mere 22.9°C. These observations suggest that the localized thermal effect induced by the photothermal conversion membrane device effectively minimizes energy loss during the heating of bulk water, thereby substantially augmenting the production of solar water vapor.

**Figure 12:**
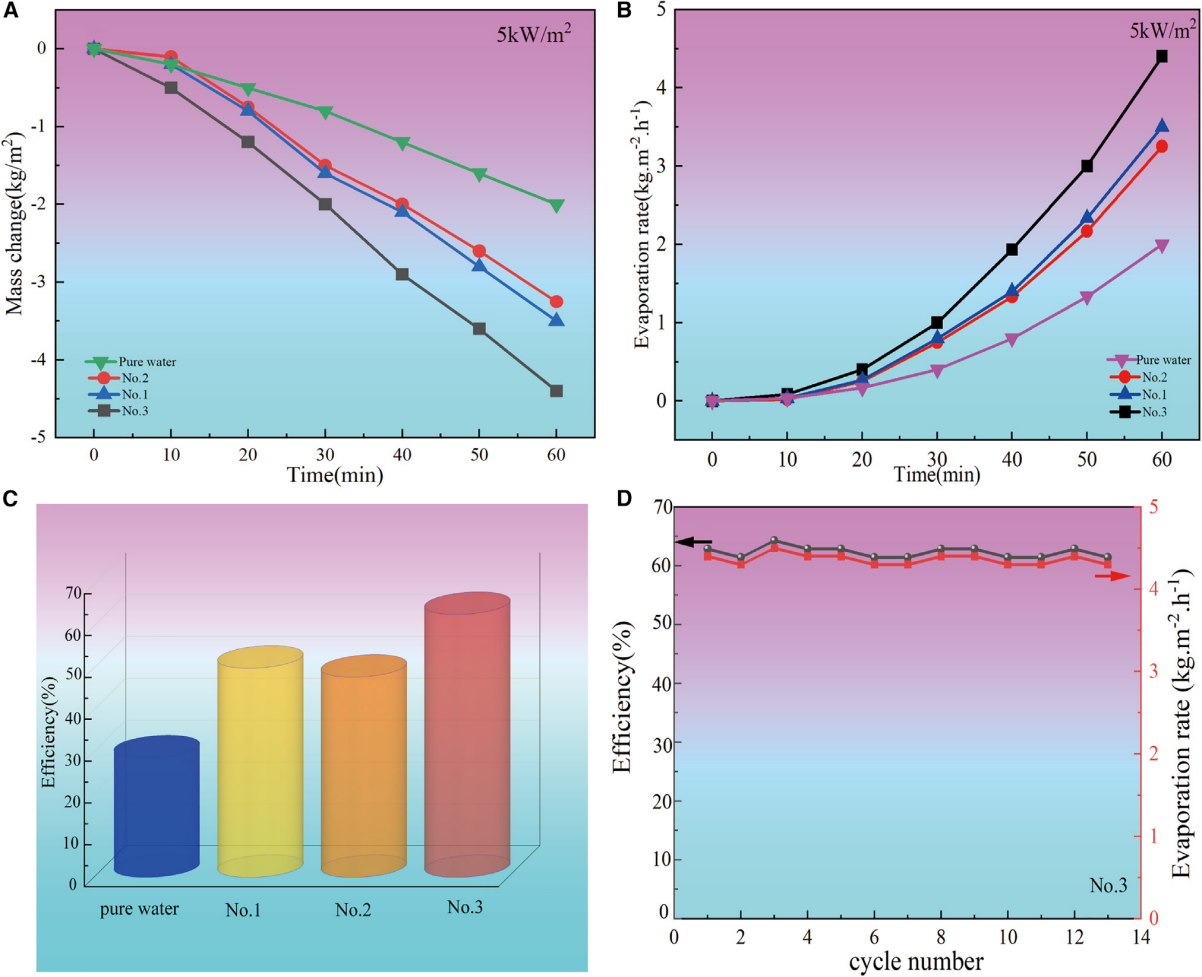
Performance evaluation of black titanium-based materials for solar-driven water evaporation under various conditions (A) Evaporation water weights against time of as-prepared black titanium-based materials prepared under different conditions under the 5 kW m^−2^ solar illumination; (B) Evaporation rate against time of as-prepared black titanium-based materials prepared under different conditions; (C) Evaporative efficiency of as-prepared black titanium-based materials prepared under different conditions; (D) Evaporation cycle performance of the as-prepared black titanium-based materials.

The solar vapor evaporation rate ($\bar{m}$) is defined as $\bar{m}=dm/Adt$, Where $\bar{m}$ is the steam evaporation rate, *m* is quality of evaporated water, *A* is surface area of light-to-heat conversion materials, *t* is times. As shown in Figures 12A and 12B), the average evaporation rates of the prepared No.1, No.2, No.3 samples and pure water were 3.25, 3.5, 4.4 and 2 kg m^−2^·h^−1^ under 5 kW m^−2^ illumination, respectively. No.3 sample has the highest evaporation rate, which is 2.2 times higher than the evaporation of pure water.

The solar steam evaporation efficiency (η) is defined as $\eta =\bar{m}h_{LV}/C_{opt}P_{0}$^34^*,* where *η* is the steam evaporation efficiency, $\bar{m}$ is the water evaporation rate under solar illumination, which can be calibrated with the dark evaporation rate, *h*_LV_ is the total enthalpy of sensible heat (315 kJ/kg, from 25°C to 100°C with specific heat of 4.2 kJ/(kg°C))and phase change of liquid to water (2256 kJ/kg),^35^ *C*opt is the optical concentration, and *P*0 is the solar illumination (1 kW m^−2^). As shown in Figure 12C, the steam evaporation efficiencies of the prepared No.1, No.2, No.3 samples and pure water were 50%, 48%, 63% and 29% under 5 kW m^−2^ illuminations.

As outlined in Table 3, a comparative analysis of the evaporation rates and efficiencies of diverse titanium-based interface evaporators is provided. It is noteworthy that the steam evaporation rate and efficiency of the black titanium-based material (No.3) developed in this research endeavor closely rival those of the Ti_2_O_3_+PVA photothermal conversion material when subjected to an irradiance equivalent to 5 kW·m^-2.^^10^

**Table 3:** Evaporation rate and efficiency of different titanium-based interface evaporators

Interface evaporator structure	Photothermal material	Incident light conditions	Evaporation efficiency(η)	Evaporation rates (kg/m^2^·h)
2D	Black TiO_x_+S S Mesh^27^	1sun	50.3%	0.80
2D	Al-Ti-O+PVDF^36^	1sun	77.5%	1.24
2D	λ-Ti_3_O_5_^28^	1sun	68.3%	1.64
2D	Submicron Ti_4_O_7_^33^	1sun	36.5%	0.53
3D	TiN+CW^19^	1sun	80%	–
3D	TiN+AAO^21^	1sun	92%	–
3D	λ-Ti_3_O_5_+PVA^28^	1sun	95.9%	6.09
3D	Ti_2_O_3_+PVA^10^	1sun	–	1.32
3D	Ti_2_O_3_+PVA^10^	5sun	–	5.03
This work	Black TiO_2_+PVA	5sun	50%	3.25
This work	(Ti_4_O_7_+Ti_5_O_9_)+PVA	5sun	63%	4.4

The stability of the material is a key factor in practical applications. We tested the No.3 sample thirteen times and the results showed in Figure 12D that No.3 sample offers good cycle stability. The experimental results indicate that the mean water evaporation rate is 4.36 ± 0.06 kg m^−2^·h^−1^, with a mean efficiency of water evaporation of 62.30% ± 0.93%.

In conclusion, the nano-black titanium-based material, crafted with Ti_4_O_7_ and Ti_5_O_9_ serving as the dominant phases and utilizing nanoscale rutile TiO_2_ as the foundational raw material, demonstrates unparalleled photothermal conversion efficiency and water evaporation proficiency. This advanced nano-black titanium material boasts the extraordinary capability to capture the full spectrum of solar radiation, while its nanoscale characteristics further elevate the photothermal conversion efficiency. Moreover, we have fabricated nano-black titanium-based thin film devices and rigorously tested their impressive efficiency and stability in generating solar steam. These results underscore the vast potential of these materials for innovative applications in seawater desalination and purification processes.

### Thermal performance of black titanium-based materials under natural light

**Figure 13:**
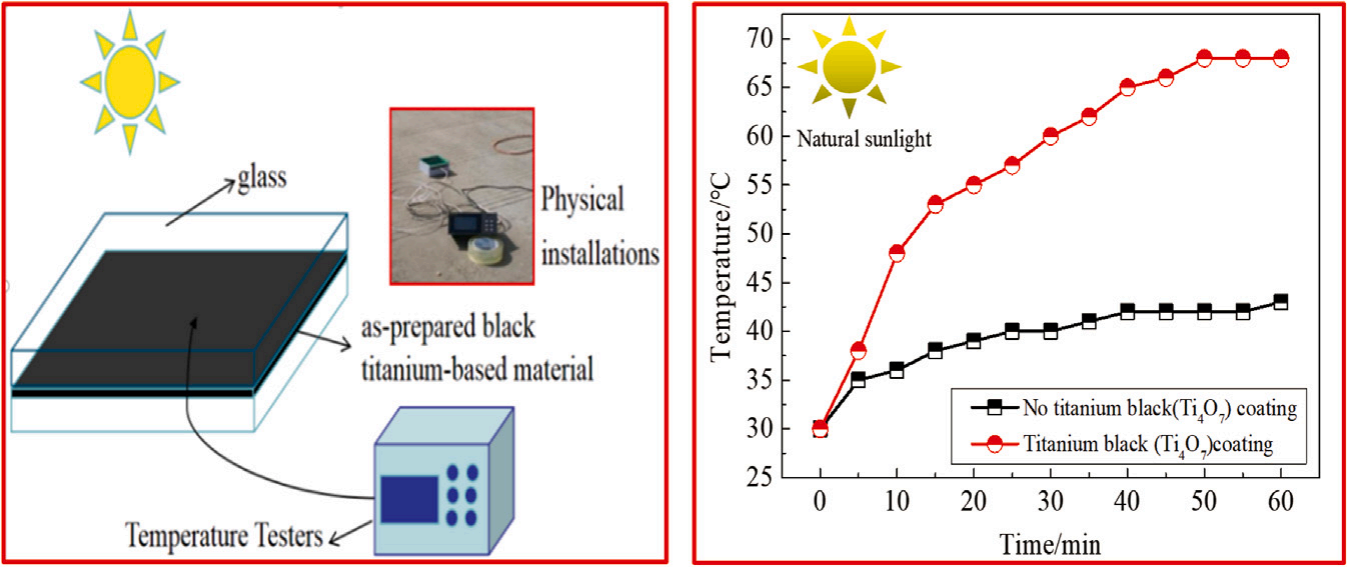
Temperature change with time of the prepared black titanium-based material under natural light conditions

We have conducted an extensive investigation into the heat-collecting performance of black titanium-based materials under natural lighting conditions. As illustrated in Figure 13, the graph depicts the temporal temperature variation of the prepared black titanium-based material (No.3) when subjected to natural light. Within a span of 50 min under sunlight exposure, the surface temperature of the black titanium-based material surged swiftly to reach 70°C. Conversely, the surface temperature of an uncoated steel sheet, serving as a comparison, lingered at a significantly lower temperature of 40°C. This observation hints at the remarkable heat-collecting efficiency of the developed black titanium-based materials when exposed to natural light.

**Figure 14:**
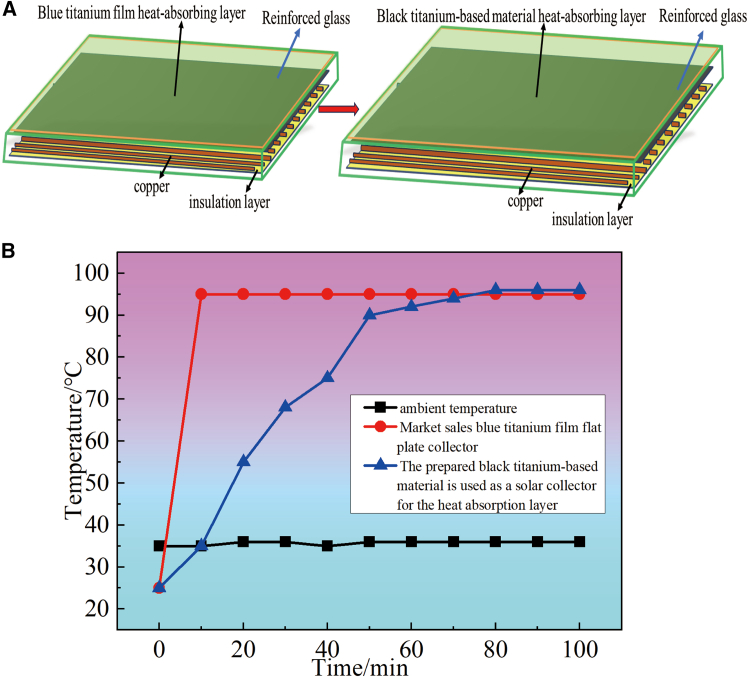
Heat collection performance of the prepared black titanium-based material and the blue titanium film flat plate collector (A) Schematic diagram of a flat-plate solar collector with heat-absorbing coatings of blue titanium film and black titanium-based material；(B) Experimental outcomes concerning the heat-collecting capabilities of both the prepared black titanium-based material and the blue titanium film flat-plate collector.

Two flat-plate collectors, featuring blue titanium foil, were acquired from the market. The structural schematic of these collectors is depicted in Figure 14A. Following the grinding process, the surface of the collectors was uniformly coated with the specially prepared black titanium-based material, which served as the heat-absorbing layer over the blue titanium film. The experimental outcomes concerning the heat-collecting capabilities of both the prepared black titanium-based material (designated as No.3) and the blue titanium film flat-plate collector are illustrated in Figure 14B. Notably, the water temperature within the flat-plate collector tube coated with the black titanium-based heat-absorbing layer can attain 95°C, mirroring the performance of commercially available blue titanium foil collectors. However, it is worth mentioning that the commercial blue titanium foil collector achieves this temperature more rapidly, requiring just 5 min to reach 95°C.These findings suggest that the black titanium-based material developed through this methodology can effectively function as a heat-absorbing layer in flat-plate collectors. This innovation significantly diminishes the cost associated with flat-plate collectors and holds promising potential for widespread market application.

### Conclusions

Black titanium-based materials with varying phases have been successfully synthesized through the aluminothermic reduction method by rationally adjusting process parameters, without the need for a vacuum or inert gas atmosphere. Black TiO_2_ was synthesized by modulating the oxygen vacancy defects on the surface of TiO_2_ at a roasting temperature of 650°C. In contrast, the Magneli phase titanium suboxide, Ti_n_O_2*n*-1_ (specifically Ti_4_O_7_ and Ti_5_O_9_), was produced at a higher roasting temperature of 950°C.

The prepared black titanium-based materials demonstrate exceptional full-spectrum solar energy absorption capabilities, achieving an overall solar absorption efficiency of 65.7% and a photothermal conversion efficiency of 87.5% for the black TiO_2_. The Magneli phase titanium oxides (Ti_4_O_7_ and Ti_5_O_9_) synthesized from rutile TiO_2_ exhibited an impressive overall solar absorptivity of 83.4% along with a photothermal conversion efficiency of 93.8%.

As a photothermal conversion material for interfacial evaporation of water, the Magneli phase titanium oxide (Ti_4_O_7_ and Ti_5_O_9_), derived from nanorutile TiO_2_ raw materials, exhibits an evaporation rate of 4.4 kg m^−2^·h^−1^ and an efficiency of 63% under light conditions of 5 kW/m^2^. These results indicate significant potential for applications in seawater desalination and wastewater purification. However, further design and optimization of the interfacial evaporator structure are necessary to achieve higher rates and efficiencies in evaporation.

As a photothermal conversion material for flat plate collectors to heat water, the black titanium-based material exhibits collector performance on par with commercially available blue titanium film, with potential as a flat plate collector photothermal conversion material and a large market application prospect in this field.

### Limitations of the study

The photothermal water evaporation performance of black titanium-based materials has primarily been evaluated under laboratory conditions, where parameters such as light intensity and temperature differ markedly from those encountered in real-world applications. Consequently, these controlled settings cannot fully replicate the complex and dynamic environmental conditions present in practical scenarios. Therefore, the actual performance and stability of black titanium-based materials in field applications may deviate from laboratory results. Further verification is required to assess their performance and stability under real-world conditions.

## Resource availability

### Lead contact

Further information and requests should be directed to and will be fulfilled by the lead contact, Jun Li (lidejun163@126.com).

### Materials availability

This study did not generate new materials.

### Data and code availability

• All data can be obtained from the lead contact, provided the request is reasonable.
• This paper does not report original code.
• Any additional information required to reanalyze the data reported in this paper is available from the lead contact upon request.

## Acknowledgments

This study was financially supported by Project of Sichuan Province Key Laboratory of Higher Education Institutions for Fine Chemical Additives and Surfactant (2023JXY01); Project of Application and Solar Technology Integration Sichuan Provincial Key Laboratory of University Program (SN240104); Project of Vanadium and Titanium Resource Comprehensive Utilization Key Laboratory of Sichuan Province (2024FTSZ04); Sichuan Province Key Laboratory of Higher Education Institutions for Comprehensive Development and Utilization of Industrial Solid Waste in Civil Engineering (SC-FQWLY-2021-Z-08); Project of Pan xi Strategic Resource Innovation and Development (LB-SK-HT23-0432); Project of Sichuan Provincial Key Laboratory of Material Corrosion and Protection (2022CL31); Project of Key Laboratory of Green Chemistry of Sichuan Institutes of Higher Education, LYJ2102).

## Author contributions

L.J. designed and supervised this work and drafted the manuscript. L.J., W.E.H., and H.J. prepared and characterized the samples. X.Z., P.W.J., L.X., and L.H. performed the experiments and data analysis. All authors viewed and commented on the manuscript.

## Declaration of interests

The authors declare that they have no competing interest.

## STAR★Methods

### Key resources table

**Table:** 

REAGENT or RESOURCE	SOURCE	IDENTIFIER
**Chemicals, peptides, and recombinant proteins**		

Anatase titanium dioxide	Sichuan Province Excellence Vanadium and titanium Co., Ltd.	MA-55
Rutile titanium dioxide	Longbai Group Co., Ltd	R-996
Aluminum powder	Jiangsu tian yuan metal powder co., Ltd.	FLQT-4
Polyvinyl alcohol	Shanghai Darui Fine Chemicals Co., Ltd	9002-89-5

**Software and algorithms**		

Origin	OriginLab Corporation	https://www.originlab.com/

**Other**		

Handheld infrared emission thermal camera	Shanghai Pumeng Optoelectronic Technology Co., Ltd	FLIRE4
500W long xenon lamp	Beijing Newbit Technology Co., Ltd	https://www.bjnbet.com/gd/2011/1104/169.html

### Experimental model and study participant details

No experimental models are used in this paper.

### Method details

#### Preparation of black titanium-based materials

50g of TiO_2_ powder was mixed with 10g of Al powder and placed in an alumina crucible. B_2_O_3_ powder was melted in a muffle furnace at 950°C for 10 minutes and applied to the surface of the mixed raw material. The black titanium-based materials were prepared after calcining the mixed raw material in a muffle furnace at 650°C and 950°C for different times. Finally, the pure black titanium material was obtained after excess hydrochloric acid was used to remove excess Al from the sample.

#### Sample characterization

The physical phase characterization of the prepared samples was carried out using a D8 advance XRD diffractometer. The surface micro-morphology of the samples was observed using a regulus 8100 field emission scanning electron microscope (SEM). The UV-Vis absorption spectra of the samples were determined using a UV-Vis spectrophotometer (D-8PC).

#### Evaluation of photothermal conversion performance

A 500W xenon lamp light source was used as the analog light source during the experiments and combined with a FLIRE4 handheld infrared emission thermal camera for temperature measurement and photography.

##### Photothermal conversion efficiency testing

5g of black titanium base material was placed in a stainless steel mold and pressed into a 36mm diameter disc under 10MPa pressure by cold press molding, and the samples were irradiated with a simulated light source of 5kW/m^2^ to test the photothermal conversion efficiency.

##### Evaporative water efficiency testing

10g of PVA powder is added to 100ml of deionized water that has been heated to 80°C in a water bath. To fully dissolve the PVA powder, turn on the stirring blade. 5g of black titanium-based material should be evenly distributed in 10ml of distilled water before being added to 10ml of PVA solution. The uniformly combined solution was transferred into a culture dish and dried at 80°C for 1 hour to create a photothermal conversion film. The surface temperature of the black titanium-based material changes with time under the irradiation of a 500W xenon lamp light source. The prepared black titanium-based photothermal conversion material was floated on the surface of a beaker filled with pure water, and the water evaporation efficiency under light conditions was tested. The schematic diagram is shown in Figure 1.

### Quantification and statistical analysis

The grain sizes are statistically estimated from the SEM images using the Nano Measurer 1.2 software. XPS spectra deconvolution and quantification is carried out using the Casa XPS analysis software. Statistical analysis of data was performed using Excel (Microsoft) and Origin (Origin Lab).
